# Hepatitis B virus virion secretion is a CRM1-spike-mediated late event

**DOI:** 10.1186/s12929-022-00827-w

**Published:** 2022-06-21

**Authors:** Pei-Yi Su, Shin-Chwen Bruce Yen, Ching-Chun Yang, Chih-Hsu Chang, Wen-Chang Lin, Chiaho Shih

**Affiliations:** 1 Graduate Institute of Medicine, College of Medicine, Kaohsiung Medical University, No.100, Shih-Chuan 1st Road, Sanmin, 80708 Kaohsiung, Taiwan; 2 Institute of Biomedical Sciences, Academia Sinica, Taipei, Taiwan; 3 Graduate Institute of Microbiology, College of Medicine, National Taiwan University, Taipei, Taiwan

**Keywords:** Hepatitis B virus (HBV), HBV core protein (HBc), HBc capsids, CRM1 (chromosome region maintenance 1), Spike, Virion secretion, Naked capsids, Microtubule, Proximity ligation assay (PLA), Therapeutic treatment

## Abstract

**Background:**

Hepatitis B virus (HBV) is a major human pathogen worldwide. To date, there is no curative treatment for chronic hepatitis B. The mechanism of virion secretion remains to be investigated. Previously, we found that nuclear export of HBc particles can be facilitated via two CRM1-specific nuclear export signals (NES) at the spike tip.

**Methods:**

In this study, we used site-directed mutagenesis at the CRM1 NES, as well as treatment with CRM1 inhibitors at a low concentration, or CRM1-specific shRNA knockdown, in HBV-producing cell culture, and measured the secretion of various HBV viral and subviral particles via a native agarose gel electrophoresis assay. Separated HBV particles were characterized by Western blot analysis, and their genomic DNA contents were measured by Southern blot analysis. Secreted extracellular particles were compared with intracellular HBc capsids for DNA synthesis and capsid formation. Virion secretion and the in vivo interactions among HBc capsids, CRM1 and microtubules, were examined by proximity ligation assay, immunofluorescence microscopy, and nocodazole treatment.

**Results:**

We report here that the tip of spike of HBV core (HBc) particles (capsids) contains a complex sensor for secretion of both HBV virions and naked capsids. HBV virion secretion is closely associated with HBc nuclear export in a CRM1-dependent manner. At the conformationally flexible spike tips of HBc particles, NES motifs overlap extensively with motifs important for secretion of HBV virions and naked capsids.

**Conclusions:**

We provided experimental evidence that virions and naked capsids can egress via two distinct, yet overlapping, pathways. Unlike the secretion of naked capsids, HBV virion secretion is highly CRM1- and microtubule-dependent. CRM1 is well known for its involvement in nuclear transport in literature. To our knowledge, this is the first report that CRM1 is required for virion secretion. CRM1 inhibitors could be a promising therapeutic candidate for chronic HBV patients in clinical medicine.

**Supplementary Information:**

The online version contains supplementary material available at 10.1186/s12929-022-00827-w.

## Background

Hepatitis B virus (HBV) is a major human pathogen [1]. As a small enveloped DNA virus, HBV genome contains four major open reading frames, encoding the envelope protein (HBV surface antigen, HBsAg), core protein (HBc), polymerase (pol), and X protein (HBx) [2]. Although current HBV vaccine is effective, curative treatment is still needed to completely eradicate the virus from most chronic HBV carriers [3]. HBc is known to have multiple functions [4]. For example, it can form capsid particles for pregenomic RNA (pgRNA) encapsidation and reverse transcription. Wild type HBV (WT-HBV) preferentially secretes virions containing mature genomes with double-stranded relaxed-circle (RC) viral DNA [5]. In contrast to WT-HBV, HBc variants 97L in patients with chronic liver disease, could preferentially secrete virions containing immature genomes of single-stranded (SS) linear DNA in cell culture and the mouse model [6–10].

**Fig. 1:**
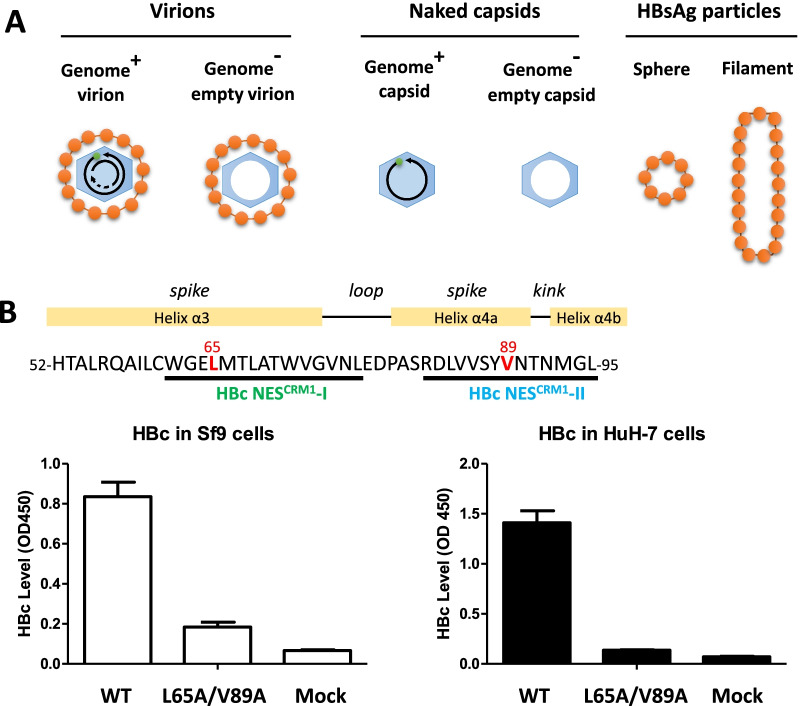
Lack of HBc secretion of an HBc double mutant L65A/V89A in human hepatocytes and insect cells. **A** A cartoon illustration of secreted HBV particles, including virions, naked capsids, and HBsAg particles. Left panel: genome-containing and genome-free empty virions contain both surface antigen and core proteins; middle panel: genome-containing and genome-free naked capsids contain no surface antigens; right panel: HBV surface antigen particles contain no core protein. **B** Upper panel: Two CRM1-dependent nuclear export signals (HBc NES^CRM1^-I and NES^CRM1^-II) were mapped to the tip of the spike of HBc particles [29]. The double mutations L65A/V89A at NES I + II are shown in red color. Lower panel: No secreted naked capsids were detected by ELISA in the media of Sf9 insect cells transfected with an HBc mutant L65A/V89A expression vector (left panel). Nor can any naked capsids be detected in the media of HuH-7 human hepatocytes transfected with an HBV replicon (right panel). The ELISA values were calculated from three independent repeat experiments. WT: wild type HBV. Mock: un-transfected cells

In addition to genome-containing virions, genome-free empty virions are also present in patients and in hepatocyte cell culture [7, 11–17]. As illustrated in Fig. 1A, virions (42 nm) are enveloped capsids assembled from HBc core protein. In contrast, HBsAg particles (22 nm) contain no core protein, while naked capsids contain no HBsAg envelope. Altogether, these various viral and subviral particles represent a common and complex secretion profile of extracellular HBV. It remains to be investigated whether these highly diverse particles of various size, shape, and compositions share the same or different egress mechanisms. Cellular host factors involved in HBV particle release had been studied, including the machinery of Endosomal Sorting Complex Required for Transport (ESCRT) [18–23]. It is anticipated that identification of new host factors involved in the release of various HBV particles could shed new light on the mechanism of HBV virion morphogenesis and secretion (Fig. 1A).

Nuclear import and export of HBc core protein and particles have been shown to depend on the arginine-rich domain (ARD) at the cytoplasmic tail of HBc [24, 25]. A cellular NXF1-p15 machinery was able to mediate the nuclear export of HBc and pgRNA [26]. By homokaryon analysis, we demonstrated that HBc is a shuttling protein translocating rapidly between nucleus and cytoplasm [24, 26]. CRM1 (chromosome region maintenance 1, exportin1, Xpo1) is a major receptor for the export of protein cargos out of the nucleus [27]. CRM1 machinery can recognize leucine-rich nuclear export signals (NES) [28]. Recently, we demonstrated that the CRM1 machinery could mediate nuclear export of HBc capsids containing encapsidated viral RNA [29]. Two CRM1 NES were identified in the spike tip of HBc capsids. NES mutations or treatment with CRM1 inhibitors strongly arrested HBc capsid particles in the nucleus. CRM1 and NXF1 machineries mediated nuclear export of HBc particles independently.

To date, nuclear export of HBc capsids and virion secretion via envelopment of HBc capsids appear to be two independent events temporally distant from each other. In our current study, at the spike tip of HBc capsid particles, we identified overlapping essential motifs for HBV virion secretion and naked capsid secretion. Coincidentally, these motifs important for HBV egress also overlapped with the CRM1-mediated NES. CRM1 inhibitors blocked the secretion of HBV virions, but not naked capsids. Similarly, treatment with an inhibitor for microtubule polymerization also blocked virion, but not naked capsid, secretion. Therefore, major routes for secretion of virions and naked capsids must be different. Surprisingly, CRM1-mediated nuclear export of HBc capsids appeared to be tightly coupled with virion secretion. It is tempting to speculate here that some of the mature capsids with RC DNA genome, or empty capsids with no DNA genome, do not necessarily bud into the ER/Golgi in the cytoplasm for envelopment and virion secretion. Instead, some of these mature capsids ready for secretion could indirectly reach the perinuclear ER/Golgi en route the nucleus. Microtubule machinery could then relay with the CRM1 nuclear export machinery for the egress of virions from the cytoplasm to the extracellular compartment. It has been known for decades that CRM1 plays a major role in nuclear transport. We report here an unexpected finding that CRM1 plays a crucial role in HBV virion secretion.

## Methods

### Cells and transfection

HuH-7 hepatocyte cell line was obtained from Dr. M. Lai at Academia Sinica, Taiwan [30]. Cells were cultured and maintained as described previously [24]. PolyJet (SignaGen) was used for HuH-7 DNA transfection. Insect cells Sf9 (*Spodoptera frugiperda 9*) were purchased from BCRC (Bioresource Collection and Research Center, Taiwan; ATCC number: CRL-1711). Cellfectin II (Gibco) was used for Sf9 transfection. Baculovirus-expressed HBc capsids were prepared as previously described [17].

### Plasmids

Plasmid pCHT-9/3091 contains a 1.1mer HBV genome (*ayw*) under a CMV promoter [31]. HBc alanine-substitution mutants in pCHT-9/3091 were generated at amino acids L65, L68, V72, V74, L76, L84, V85, V86, V89, M93, or L95 by Site-Directed Mutagenesis (Agilent Technologies, U SA). The baculovirus plasmids (pFastBac1 vector) containing WT and mutant HBc were generated with the Bac-to-Bac baculovirus expression system (Thermo Fisher Scientific, Waltham, MA, USA), and amplified in Sf9 insect cells. Leptomycin B resistant CRM1 mutants, mtCRM1-C528S–mCherry and HA-mtCRM1 C528S, were kindly provided by Dr. Urs F Greber and Dr. Ralph H Kehlenbach [32, 33].

### Antibodies and inhibitors

Antibodies used here are from different sources: anti-tubulin (GeneTex), anti-lamin B1 (GeneTex), anti-CRM1 (Sigma), anti-HBV capsid (Hyb-3120) (Institute of Immunology, Japan) [34] and rabbit anti*-HBc* [24, 26]. The SINE (Selective Inhibitor of Nuclear Export) compound Verdinexor (KPT-335) was purchased from Selleck Chemicals, USA, and was added during transfection.

### Accession numbers

GenBank accession numbers: CRM1 (XPO1) 7514/O14980; hepatitis B virus (*ayw*) V01460.

### In situ proximity ligation assay (PLA)

The in situ PLA assay was performed as described [35]. HBV transfected HuH-7 cells were fixed at 48 h post-transfection. For HBc/microtubulin interaction, sample slides were incubated at room temperature for 1 h with HBc specific rabbit polyclonal antibody (1:1000) [24, 26] andα-tubulin specific monoclonal antibody DM1A (1:200, GeneTex). For CRM1/microtubulin interaction, rabbit anti-CRM1 antibody (1:200, Sigma) and anti-αtubulin DM1A (1:200, GeneTex) were used. For CRM1/HBc capsid interaction, mouse monoclonal anti-HBV capsid (Hyb-3120) (1:200, Institute of Immunology, Japan) was used [24, 26]. Samples were processed using the Duolink® In Situ Red Starter kit Mouse/Rabbit (DUO92101, Sigma), according to the manufacturer’s instructions. The Z-stack PLA Dots signal images were collected by using a Zeiss LSM700 stage confocal microscope system (Carl Zeiss, Jena, Germany) and analyzed by the MetaMorph analysis software (Molecular Devices). The number of PLA dots per cell is shown in the graph of Fig. 6B–D. The statistics was analyzed by Excel two-tails Student’s* t* test.

### Immunofluorescence assay and statistical analysis

Briefly, HBV genome was transfected into HuH-7 cells. Cells were fixed 48 h post-transfection. IFA analysis was performed as described in detail elsewhere [24, 26]. Three major patterns of HBc subcellular distribution (Cy > Nu, Nu > Cy, Nu&Cy) were scored. Cy > Nu: predominant cytoplasmic HBc; Nu > Cy: predominant nuclear HBc; Nu&Cy: HBc in both compartments. Raw data of HBc statistical distribution are shown in Additional file 1: Fig. S4. Statistics were based on the software of Excel student’s* t* test.

### Cytotoxicity assay

HuH-7 cells were transfected with wild type HBV in the absence or presence of SINE compounds (0.1 ~ 2.5 μM). Cytotoxicity was assayed by using AlamarBlue™ Cell Viability Reagent (Thermo Fisher Scientific Inc.) and Cell Counting Kit-8 (CCK-8, Dojindo Molecular Technologies, Inc.).

### Northern and southern blot analyses

HBc capsid particles were prepared using the PEG precipitation method [17]. Experimental procedures for the extractions of encapsidated viral RNA and DNA, as well as Southern and Northern blot analyses, are as described previously [17]. DIG-labeled-full-length HBV DNA was used as a probe. Signal intensity was measured by a densitometry program ImageJ (NIH).

### Knockdown by shRNAs

Lentivirus shRNAs for CRM1 knock down (ShCRM1: TRCN0000152787, TRCN0000150975, TRCN0000338401, and TRC1.Scramble) were purchased from RNAiCore, Academia Sinica, Taiwan.

### Other experimental procedures

ELISA was performed as described in a previous report [24].

### Quantification and statistical analysis

Student’s t tests were performed using GraphPad Prism and Excel software. The methods of quantification and statistical analysis in each experiment were as described above in the “Method details” Section.

### Data availability

All data generated or analyzed in this study are included in this published article.

## Results

### History and rationale

Previously, we identified two CRM1-mediated NES nuclear export signals at the spike tip of HBc capsids [29] (upper panel, Fig. 1B). To further investigate this phenomenon, we overexpressed HBc capsids by using the insect sf9 cells. Here, as shown in the lower panel of Fig. 1B, when we transfected a WT-HBc expression vector into insect cells sf9, we observed strong signals of HBeAg/HBc in the medium by the ELISA assay. This is expected because empty core particles are known to be secreted into the medium of sf9 cells [36]. Similarly, when we transfected a WT-HBV replicon into HuH-7 hepatocytes, naked core particles can be detected in the medium [17, 22]. However, to our surprise, little core protein signal was detected when transfected with an HBc NES mutant L65A/V89A (Fig. 1B). To follow up this issue, we established an agarose gel electrophoresis assay, in combination with Western, Southern and Northern blot analyses, to examine the extracellular HBV secretion profiles of various viral and subviral particles (Additional file 1: Fig. S1A). As a control, intracellular capsid assembly, as well as capsid-associated viral DNA and RNA, were also monitored (Additional file 1: Fig. S1B).

### A hub of multiple capsid traffick signals clustering at the spike tip

**Fig. 2:**
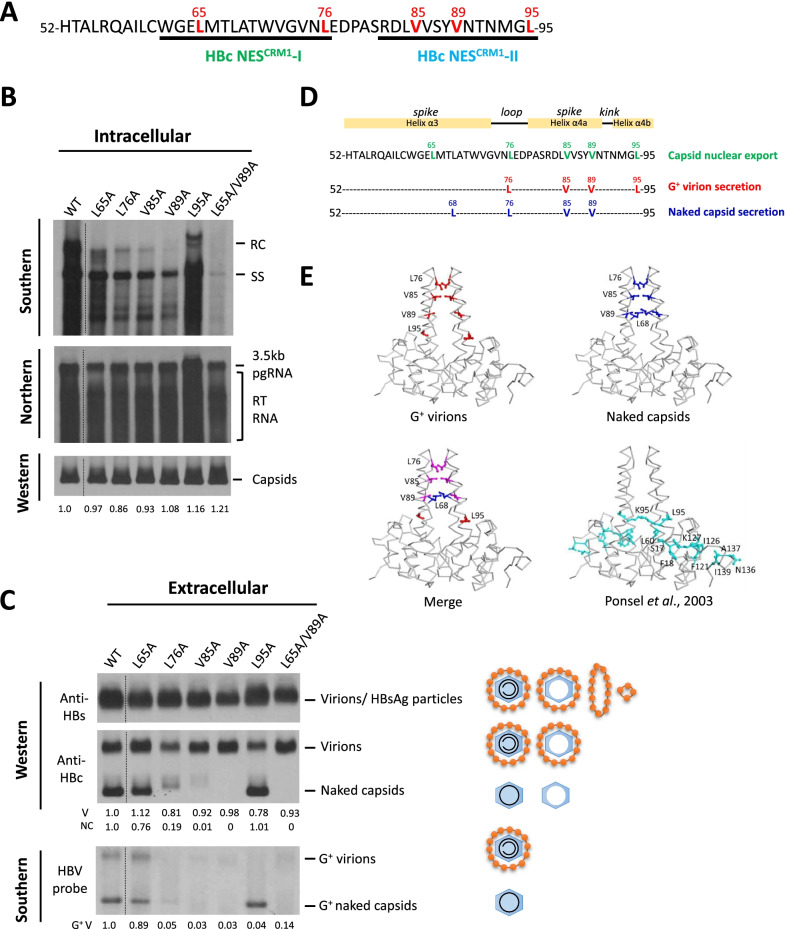
The tip of the spike at HBc 76–89 is required for secretion of both genome-containing virions and naked capsids. **A** Site-directed mutations were introduced into amino acid L65, L76, V85, V89 and L95 (red colored) of NES-I and NES-II. These mutants accumulated HBc in the nucleus as described [29]. **B** Intracellular core-associated RNA and DNA genomes were analyzed by Northern blot and Southern blot. None of these HBc mutants are defective in packaging the 3.5 kb full-length pgRNA. Except L95A, mutants L65A, L76A, V85A, V89A exhibited some minor defect in RC DNA synthesis. All results shown in (**D**) represent one of three independent repeat experiments. The vertical dotted line indicates splicing of data from the same gel. **C** Extracellular HBV particles were analyzed by the method illustrated in Additional file 1: Fig S1A. Single mutation L76A, V85A, V89A inhibited secretions of both genome-containing virions and naked capsids. However, mutation L95A inhibited only genome-containing virion secretion, but no effect on naked capsid secretion. All results shown in (**B**) represent one of three independent repeat experiments. The vertical dotted line indicates splicing of data from the same gel. **D** The tip of spike is a hub of multiple trafficking signals of HBc particles. Comparisons of important amino acid residues of HBc involved in HBc nuclear export, virion and naked capsid secretions. **E** A three dimentional view (PDB code 1QGT) of HBc dimer which can assemble into an icosahedral particle. Key HBc amino acid residues involved in the secretion of particles, as highlighted in (**C**), are clustering at the tip of the spike of HBc capsids. Red: secretion of genome-containing virions. Blue: secretion of naked capsids. Merge: purple. For comparison, we included a previous map of amino acids at the bottom of the spike, which are also involved in the secretion of HBV genome-containing virions (cyan) [37]. This figure was produced using PyMOL (Schrödinger, LLC; http://www.pymol.org)

Intrigued by the results in Fig. 1B, we extended our studies from HBc mutant L65A/V89A to HBV replicons containing a single mutation in NES^CRM^-I and NES^CRM^-II (Fig. 2A). In the intracellular control experiment, except for mutant L95A, the rest of NES mutants were somewhat compromised in viral DNA synthesis (upper panel, Fig. 2B). None of these mutants are defective in the 3.5 kb pgRNA encapsidation (middle panel, Fig. 2B) and capsid assembly (lower panel, Fig. 2B and Additional file 1: Fig. S2C). In the extracellular experiment, consistent with the previous ELISA results in Fig. 1B, we detected no naked capsids in mutant L65A/V89A (last lane in Fig. 2C). Despite its normal capsid assembly (bottom panel, Fig. 2B), mutant L65A/V89A exhibited no intracellular viral DNA synthesis (top panel, Fig. 2B) and thus no genome-containing G^+^ virions and G^+^ naked capsids (lower panel, Fig. 2C). In contrast, both empty virions and HBsAg particles can be visualized in all of the NES mutants by using anti-HBc and anti-HBs antibodies, respectively (upper panel, Fig. 2C). While single mutants L76A, V85A, V89A, and L95A lost G^+^ virion secretion (lower panel, Fig. 2C), single mutant L76A, V85A, V89A (Fig. 2C) and L68A (Additional file 1: Fig. S2A and Fig. S2B) lost both G^+^ and G^−^ naked capsids. As summarized in Fig. 2D, mutation L95A affected only G^+^ virion secretion, but not naked capsids. In contrast, L68A affected only naked capsid, but not virion secretion (red asterisk in Additional file 1: Fig. S2C).

To compare our mutagenesis results with those in the literature, we color highlighted key residues involved in virion secretion in the context of a 3D structure of HBc dimer and hexamer (Fig. 2E; Additional file 1: Fig. S2D). Overall, key residues involved in virion secretion identified in our current study are mainly at the spike tip, while key residues from an earlier study tend to cluster as a ring-like groove around the base of the spike [37]. Except for L95, no other key residues are in common between our current study and that earlier report [37], and thus no contradiction in experimental results with each other.

### Drastic reduction in virion secretion by 0.1 μM CRM1 inhibitors

**Fig. 3:**
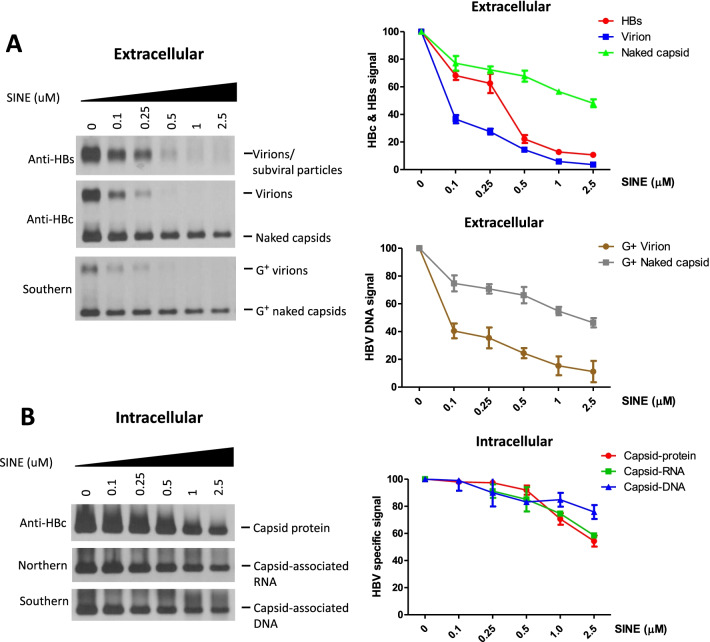
CRM1 inhibitors blocked secretion of HBV virions but not naked capsids. **A** Left panel: Secretions of WT-HBV virions and HBsAg particles were strongly inhibited by SINE compound at 0.1 μM in a dose–response curve. While virion secretion was clearly reduced when SINE compound concentration was increased, secretion of naked capsid was far less sensitive to drug treatment. Similarly, Southern blot analysis revealed that the secretion of genome-containing virions was much more sensitive to SINE compounds than the secretion of genome-containing naked capsids. Right panel: Bar graphs were plotted based on the signal intensities of HBc, HBs and HBV DNA, as measured by a densitometer and the Image J Software. The values were calculated as mean of more than three independent experiments. **B** Left panel: As a control to the extracellular secretion profiles in (**A**), we examined the total intracellular lysate for the WT-HBV capsid protein, capsid-associated RNA and DNA, in the same dose–response experiment by the SINE compound treatment. Right panel: HBV specific signals were plotted as a bar graph. The values were calculated as mean of more than three independent experiments

Because of the coincidence of the NES^CRM1^ motif with the sequence motif involved in G^+^ virion secretion (Fig. 2C), we asked if co-treatment with CRM1 inhibitors during transfection could interfere with virion secretion. In a dose–response experiment using SINE compound from 0.1 to 2.5 μM, we observed gradual reduction in total virions and HBsAg particles (upper panel, Fig. 3A). Significant reduction began at 0.1 μM and no virion was detectable after 0.5 μM. In the cytotoxicity assays in cell culture (Additional file 1: Fig. S3), no apparent cytotoxicity was detected when SINE concentration was below 0.25 μM. By Southern blot analysis (lower panel, Fig. 3A), it is clear that G^+^ virions were significantly reduced when CRM1 was inhibited by SINE treatment. Since the vast majority of secreted virions are known to be empty without a dsDNA genome [7, 11–17], the apparent reduction in the total virions must reflect an inhibition of the empty virion secretion at 0.25 μM SINE (middle panel, Fig. 3A). In contrast to the enveloped virions, secreted naked capsids (with or without a genome) were far more resistant to SINE compound, even at the high concentrations between 0.5 and 2.5 μM SINE. Unlike the previous genetic results in Fig. 2C, we noted that the levels of both HBsAg particles and empty virions were strongly reduced even at 0.25 μM concentration (top panel, Fig. 3A; see Discussion). Graphic presentations revealed that the most rapid decline of virions occurred at 0.1 μM SINE compound (bottom, Fig. 3A). The reduction in the extracellular virions and HBsAg was not caused by any deficiency in the intracellular viral replication and gene expression. As shown in Fig. 3B, intracellular capsids and capsid-associated viral RNA and DNA exhibited only moderate decrease upon drug treatment, relative to the drastic decline of secreted virions.

### Association between nuclear export and virion secretion

**Fig. 4:**
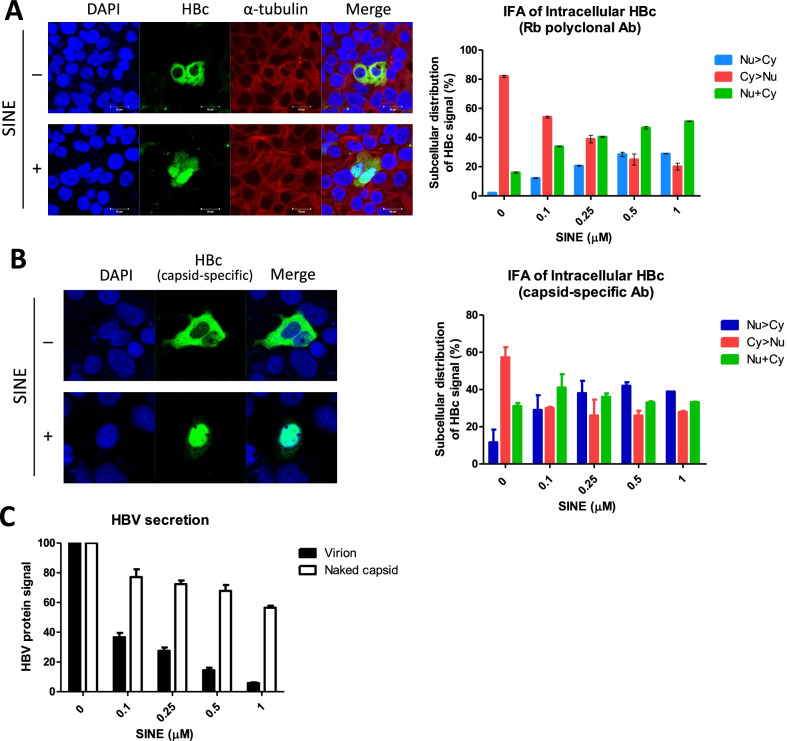
The reduction of HBV virion secretion by CRM1 inhibitors is correlated with the increased HBc nuclear accumulation. **A** HBc subcellular distribution was visualized by immunofluorescence assay (IFA) using a rabbit polyclonal anti-HBc antibody. Upon treatment with SINE compound, HBV transfected HuH-7 cells accumulated rapidly HBc in the nucleus. Three major patterns of HBc subcellular distribution (Cy > Nu, Nu > Cy, Nu&Cy) were scored [26]. The bar graph (right panel) summarizes the changing HBc distribution patterns in a dose–response experiment. Cy > Nu: predominant cytoplasmic HBc; Nu > Cy: predominant nuclear HBc; Nu&Cy: HBc in both compartments. Raw data of HBc statistical distribution are from Additional file 1: Fig. S4. **B** Similar results were obtained by using a capsid-specific mouse monoclonal antibody (Mab 3120), indicating that HBc capsid particles were accumulated in the nucleus upon drug treatment. **C** This phenomenon of HBc nuclear accumulation upon SINE compound treatment (**A** and **B** above) is strongly associated with the reduction of virion secretion, but not naked capsid secretion, in repeated dose–response studies. Raw data of this bar graph are from the Western blot of (**A**). HBc protein signals of secreted virions (solid bar) and naked capsids (open bar) in the no drug treatment experiment of (**A**), are shown here as a 100% reference control

In an earlier study, we observed very strong nuclear accumulation of HBc upon treatment with SINE compounds at 0.5–1.0 μM concentration [29]. We therefore asked if nuclear arrest of HBc protein and particles could be related or coupled to the reduced virion secretion (Fig. 3). Indeed, when 0.1 μM SINE compound was added to the transfected culture, we observed strong accumulation of HBc protein (Fig. 4A) and particles (Fig. 4B) in the nucleus by IFA. A rabbit anti-HBc protein polyclonal antibody was used in Fig. 4A and Additional file 1: Fig. S4A, while a capsid particle specific mouse monoclonal antibody (Mab3120) was used in Fig. 4B and Additional file 1: Fig. S4B. The subcellular distribution of HBc in the transfected HuH-7 population can be classified into three different patterns: nucleus-predominant (Nu > Cy), cytoplasm-predominant (Cy > Nu), and both nucleus and cytoplasm (Nu&Cy). The cell numbers of each pattern were scored under confocal microscopy (Additional file 1: Fig. S4). At the same 0.1 μM SINE concentration, significant reductions in both virion secretion (Fig. 4C) and nuclear export (Fig. 4A, B) were detected. When the SINE concentration was gradually increased, more and more HBc was arrested in the nucleus, and less virions were secreted. This inverse correlation suggests that nuclear HBc and virion secretion could be tightly coupled.

### Knockdown of CRM1 blocked virion secretion

**Fig. 5:**
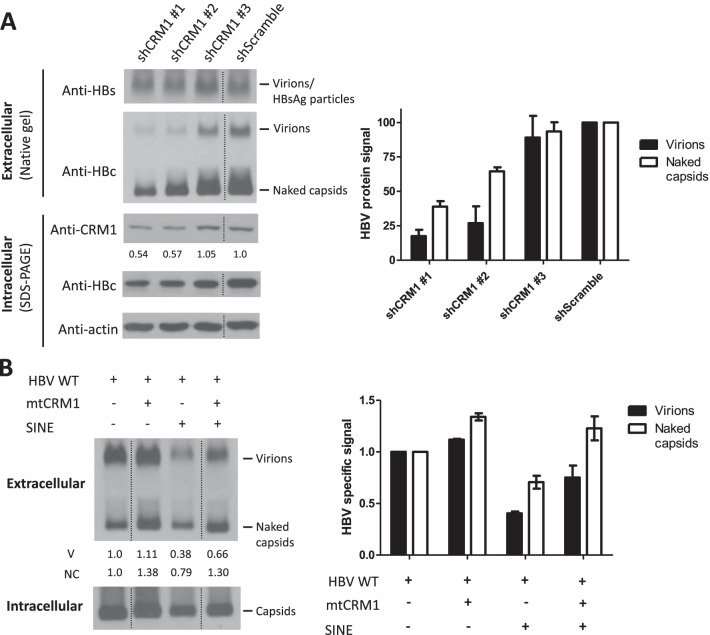
CRM1 is directly involved in HBV virion secretion. **A** In an HBV transient transfection system in HuH-7 cells, Lentivirus shCRM1 #1 and #2 significantly knocked down CRM1 expression and reduced HBV virion secretion. The bar graph (right panel) is based on the signal intensities of virions and naked capsids (left panel) using densitometry and Image J. The mean values were calculated from at least three independent experiments. shScramble: a non-specific control. The vertical dotted line indicates splicing of data from the same gel. HBc protein signals of secreted virions (solid bar) and naked capsids (open bar) in the shScramble experiment, are shown here as a 100% reference control. Both CRM1 and HBc proteins in the total intracellular lysates were assayed by SDS-PAGE. **B** The reduction of HBV virion secretion by 0.1 μM SINE compound treatment can be rescued by cotransfection with a drug-resistant mutant CRM1 expression vector (see Methods). The bar graph (right panel) is based on the signal intensities of virions and naked capsids (left panel) using densitometry and Image J. The mean values were calculated from at least three independent experiments. The vertical dotted lines indicate splicing of data from the same gel. HBc protein signals of secreted virions (solid bar) and naked capsids (open bar) in the no treatment experiment (HBV WT only), are shown here as a reference control (1.0)

In addition to the SINE treatment experiment, we performed a CRM1-specific shRNA knockdown experiment. Consistent with the SINE compound effect on virion secretion (Fig. 3A and 4C), a twofold reduction in the intracellular CRM1 protein could lead to a near fourfold reduction in virion secretion (middle panel, Fig. 5A).

### Rescue of virion secretion by a drug-resistant mutant CRM1

The lack of virion secretion in SINE-treated culture can be rescued efficiently by cotransfection with a drug-resistant mutant CRM1 expression vector (Fig. 5B) [32]. We noted that the secretion of naked capsids was rescued simultaneously. In addition, secretion of HBsAg was also rescued in the ELISA assay (Additional file 1: Fig. S5A). Similarly, nuclear accumulation of HBc (Nu > Cy) induced by SINE can be reverted to the cytoplasm-predominant pattern (Cy > Nu) after cotransfection with the drug-resistant mutant CRM1 (Additional file 1: Fig. S5B) [33]. Finally, by nucleus-cytoplasm fractionation and Western blot analysis, drug-resistant mutant CRM1 again rescued the cytoplasmic HBc level in the presence of SINE compound (Additional file 1: Fig. S5C).

### Microtubules and virion secretion

**Fig. 6:**
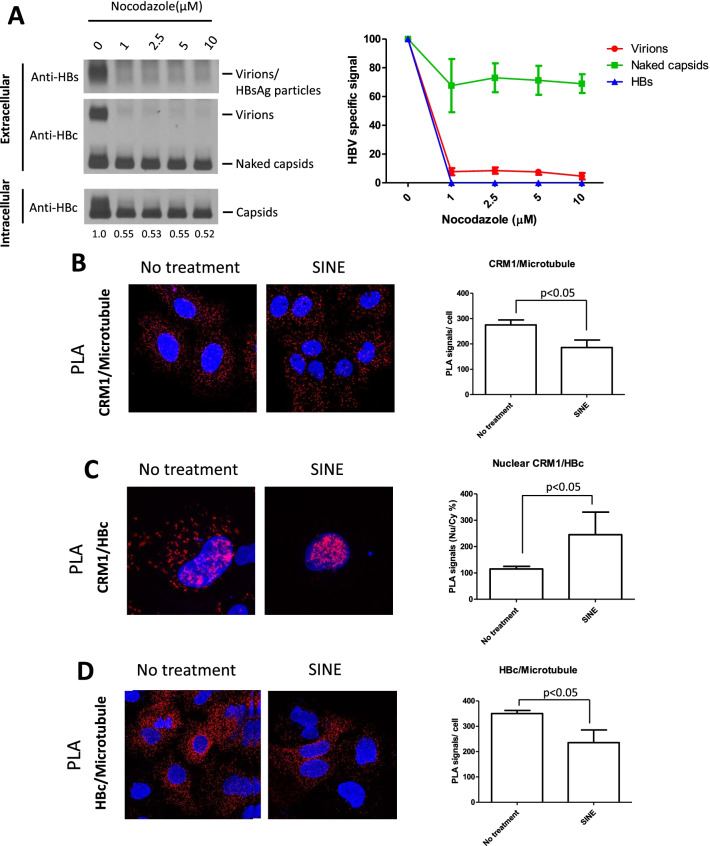
HBV virion secretion is microtubule dependent. **A** HBV virions secretion can be inhibited by Nocodazole treatment at low concentration (1 μM). In contrast, secretion of naked capsids was highly resistant to the Nocodazole treatment. The bar graph (right panel) is based on the signal intensities of virions and naked capsids (left panel) using densitometry and Image J. The mean values were calculated from three independent repeat experiments. HBV virion secretion, but not naked capsid secretion, could be microtubule-dependent. **B** Potential CRM1 and microtubule interactions were measured by detecting the immunofluorescence using the Duolink® PLA reagents (left panel). The bar graph (right panel) indicates the CRM1/Microtubule PLA interactions (< 40 nm) per cell by MetaMorph Microscopy Automation and Image Analysis Software. Respective cell numbers scored: no treatment (337 cells), SINE (385 cells). SINE compound treatment decreased the CRM1 and microtubule interactions. Student *t* test was used for statistics. **C** HBc and CRM1 interactions were measured similarly by Duolink® PLA reagents. HBc and CRM1 accumulated in the nucleus after SINE compound treatment. Cell numbers scored: no treatment (849 cells), SINE (325 cells). **D** HBc and microtubule interactions were measured by Duolink® PLA reagents. SINE compound treatment decreased the HBc and microtubule interactions. Cell numbers scored: no treatment (565 cells), SINE (413 cells)

It is known that the microtubule cytoskeleton could be involved in the intracellular trafficking of several large-sized viral particles, including nuclear targeting and capsid assembly [38–41]. We asked whether microtubules could be involved in HBV virion secretion. Nocodazole is known to inhibit microtublule polymerization [42]. When HBV-transfected culture was treated with nocodazole in a dose–response experiment (1–10 μM), secretion of virions and HBsAg particles was abolished, while intracellular capsids and extracellular naked capsids were only slightly affected (Fig. 6A). As an initial attempt to investigate the in vivo relationship between virion secretion and microtubules, we used the proximity ligation assay (PLA) to examine the in vivo interactions between CRM1, HBc, and microtubules (Fig. 6B–D). When HBV-transfected HuH-7 hepatocytes were treated with 0.25 μM SINE, the overall PLA signal between CRM1 and microtubule is mainly in the cytoplasm, and the PLA signal per cell was significantly reduced (p < 0.05) (Fig. 6B). In the case of the in vivo interaction between HBc and CRM1, SINE compounds induced striking accumulation of PLA signals in the nucleus, indicating that both CRM1 and HBc were colocalized in close proximity to each other in the nucleus (Fig. 6C). Both HBc and CRM1 are nucleocytoplasmic shuttling proteins [24, 26, 27, 43]. In the absence of SINE, both HBc and microtubules were mainly in the cytoplasm, and thus as expected, the PLA signals were almost exclusively in the cytoplasm (Fig. 6D). Upon treatment with SINE, HBc shifted from cytoplasm to nucleus leading to reduced interaction between HBc and microtubules in the cytoplasm, and thus reduced cytoplasmic PLA signals. By confocal microscopy, we also observed colocalization between CRM1, tubulin and HBc protein at the perinuclear region (Additional file 1: Fig. S6).

## Discussion

HBc capsids fold into a so-called “4-helix bundle” structure, and the tip of spike consists of a short loop sandwiched between helix α3 and helix α4 (Fig. 1B) [44–47]. Recently, we reported that the CRM1 machinery can export the RNA-containing capsids from nucleus to cytoplasm at the early phase of the HBV life cycle [29]. Two leucine-rich CRM1-specific NES were identified at the spike tip of HBc capsids. Surprisingly, as summarized in Fig. 2C, except for amino acid L65, key residues (L76, V85, V89, and L95) involved in the nuclear export of HBc particles coincided with those key residues important for genome-containing (G^+^) virion secretion. Relative to other NES mutants L76A, V85A, V89A, and L95A, mutant L65A exhibited the weakest phenotype in HBc nuclear arrest by the IFA assay (see Fig. 1E in ref 29). This might explain why mutant L65A is only slightly reduced in DNA synthesis (Fig. 2B) and perfectly normal in virion secretion (Fig. 2C). In contrast to L65A, mutants L76A, V85A, and V89A are severely defective in genome-containing virion secretion (Fig. 2C). These mutants produced appreciable amount of near full-length (shorter-than-full-length) RC DNA signal, albeit their full-length RC DNA and overall DNA synthesis were compromised (Fig. 2B). A near full-length RC DNA genome, including all signals banding above the SS DNA, is sufficient for HBV to trigger virion secretion. One good example here is mutant L65A. Although it lacked the 100% full-length RC DNA, it exhibited near full-length RC DNA (Fig. 2B), and was perfectly normal in genome-containing G + virion secretion and total (G + and G−) virion secretion (Fig. 2C). In another example, the genome maturity of mutant L95A is as normal as the WT (Fig. 2B), yet it is completely devoid of G + virion secretion (Fig. 2C). Therefore, the defect in virion secretion of these spike tip mutants is not likely to be a secondary consequence from a primary defect in RC DNA synthesis and genome maturation.

We noted that both naked capsid and virion secretions were affected simultaneously by the same NES mutations L76A, V85A, and V89A (bottom panel, Fig. 2C), by the same CRM1-specific shRNA treatment (Fig. 5A), and rescued simultaneously by the same drug-resistant CRM1 expression vector (Fig. 5B). Taken together, the frequent association between naked capsid secretion and virion secretion suggests that the secretions of virions and naked capsids are likely to share the same CRM1-mediated pathway. It is most intuitive and straightforward to assume a simple precursor-product relationship between naked capsids and secreted virions. However, if so, then in Additional file 1: Fig. S2C, the loss of naked capsids in mutant L68A and V86A (red asterisk) should have led to the reduction of virions. Exactly opposite to this prediction, we observed significantly increased secretion of virions in mutants L68A and V86A. This inverse correlation between naked capsids and virions suggests, instead, the existence of two respective secretion pathways branching out of a common upstream precursor pool of intracellular capsids. As shown in Fig. 2D, the motifs for virion secretion and naked capsid secretion are overlapping, but not identical. In Fig. 2E, amino acid L68 is highlighted in blue color and not overlapping with the red color motif for virion secretion. It is worth mentioning here that a reverse phenomenon opposite to the secretion phenotype of mutant L68A was reported previously [22]. An HGS host factor (often interacting with the ESCRT machinery) can promote the secretion of naked capsids at the expense of virion secretion. If a naked capsid precursor must undergo additional envelopment leading to virion secretion, then HGS should have stimulated the secretions of both naked capsids and virions.

Finally, the secretion of virions was much more sensitive than the secretion of naked capsids to SINE and Nocodazole treatments (Figs. 3A and 6A). The significant difference in drug sensitivity suggest again that virions and naked capsids are secreted via two different pathways. At present, we entertain this possibility that most of the arrested capsids in the nucleus are mainly mature and empty capsids, which are ready for secretion as virions via an ER-Golgi route. In contrast, the immature capsids cannot get imported into the nucleus, and thus are predominantly distributed in the cytoplasm, ready for secretion as naked capsids via an HGS-dependent, ER-Golgi independent route. In this scenario, virion secretion should be more sensitive to SINE compound than the naked capsid secretion. Indeed, in a time course experiment, we observed that immature capsids containing RNA pregenome were predominantly cytoplasmic, and mature capsids containing double-strand DNA genome were strongly accumulated in the nucleus (Ching-Jen Yang, Ching-Chun Yang, and Chiaho Shih, unpublished observation). In summary, we conclude here that naked capsids and virions egress through two different and mutually competing routes.

We envisioned the protruding spike tip as a hub of high-density antennas which can receive multiple different signals for capsid trafficking and morphogenesis. It is worth mentioning here that the spike tip is known to be highly conformationally flexible [48–53]. Conceptually, the spike tip of HBV capsids is reminiscent of a locally versatile intrinsic disorder peptide [54], which can partner with different egress machineries for different particles. It would be interesting to see whether the spike tip of other non-HBV icosahedral particles could be as versatile in sensing multiple signals.

In our earlier study, we discovered two naturally occurring HBc mutations P5T and L60V, both of which exhibited a low level of virion secretion [55]. Similarly, laboratory-engineered mutants P5G, P5W, L60G, and L60W, are competent for DNA synthesis, but failed to secrete G + virions [56]. Ponsel et al. (2003) identified eleven HBc mutations with a similar phenotype of no or low virion secretion, including L60A and L95A. These mutations form a ring-like groove around the base of the spike. Here, we identified key residues (L76, V85, V89, and L95) at the tip of the spike, which are important for genome-containing virion secretion (Fig. 2C, E).

In addition to the low-level virion secretion variant P5T and L60V, a predominant naturally occurring HBc variant 97L (I97L or F97L), exhibited an immature secretion phenotype with excessive amounts of virions containing an immature genome [6–10]. This kind of immature secretion phenotype is not consistent with the dogma of the wild type HBV which secretes only or preferentially mature genomes [5]. By changing into 17 other amino acids via site-directed mutagenesis at HBc position 97, leucine 97 (97L) is the one and only one substitution which can generate an immature secretion phenotype. In addition, a hydrophobic pocket around amino acid 97 was identified [57, 58]. The mechanism behind this immature secretion phenotype remains a mystery. Mutant F97L was shown to assemble capsids more efficiently than the WT HBV [59]. Recently, an unknown pocket-binding factor was proposed to trigger a conformational switch of capsids for envelopment and virion secretion [56, 60]. Here, it is tempting to speculate that a newly formed NES^CRM1^ with a more exposed or more flexible structural motif of clustering leucines (93- MGLK**L**RNLLWF-103; 97L underlined), could invite CRM1 to bind to the hydrophobic pocket in the center of the spike. The conformational change induced by this binding could mimic a genome maturation signal [5] and thus contribute to the immature secretion phenotype.

HBsAg specific RNAs contain a so-called post-transcriptional regulatory element (PRE) (nt 1219-1584), which can facilitate nuclear export of HBsAg specific RNAs [61, 62]. To date, it remains controversial whether PRE-mediated HBsAg RNA export is CRM-1 dependent [63] or independent (resistant to leptomycin B) [64, 65]. In Fig. 3A, we observed significant reduction of HBsAg particles at 0.1 μM SINE, and no detectable extracellular HBsAg signals at 0.5 μM. In Fig. 6E in our previous paper [29], 1.0 μM SINE treatment significantly reduced the HBsAg RNAs. However, at 0.5 μM SINE (Fig. 6F in ref 29), we detected no apparent effect on the intracellular HBsAg specific RNA by Northern blot analysis. Therefore, in our current study, we kept the SINE concentration at 0.1 ~ 0.25 μM. At this low concentration, we observed no apparent cytotoxicity (Additional file 1: Fig. S3), no appreciable effect on the intracellular viral DNA synthesis, RNA encapsidation, and HBc protein production (Fig. 3B). It is noteworthy that in Fig. 2C, while the HBsAg levels of NES mutants L76A, V85A, and V89A were normal, their G + virion secretion was almost completely blocked. Similarly, shCRM1 treatment blocked virion secretion with no significant effect on HBsAg (Fig. 5A).

**Fig. 7:**
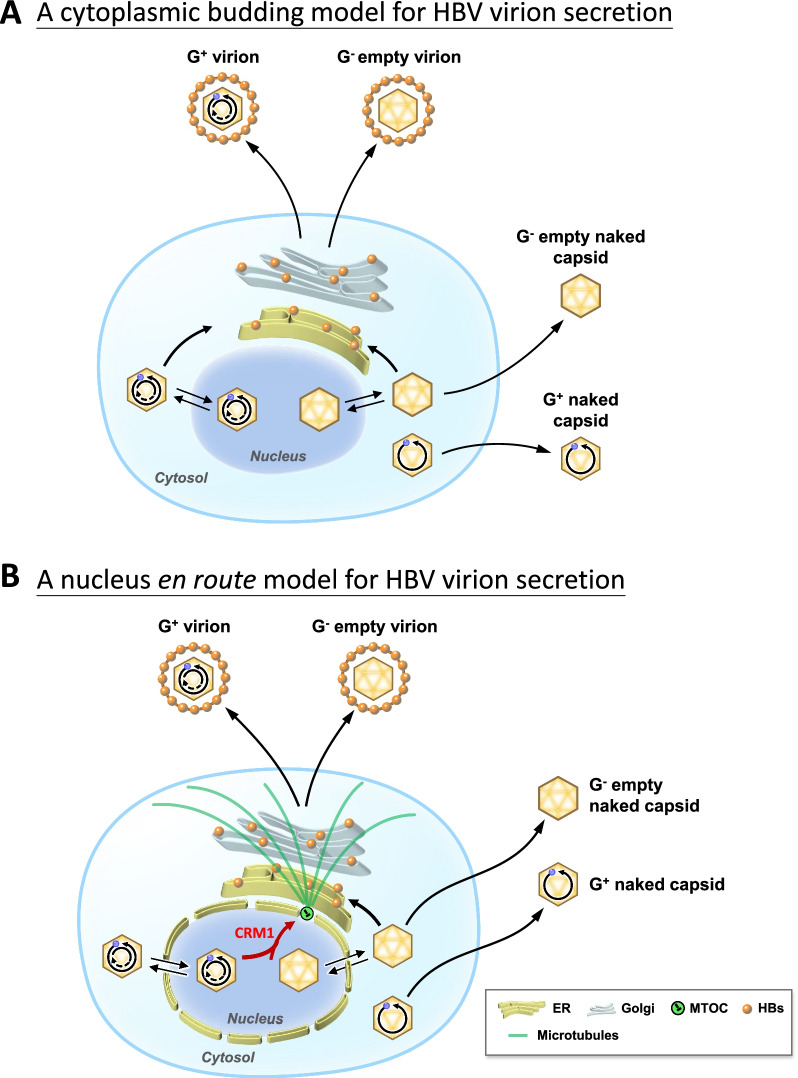
Two different models of HBV virion secretion. **A** A current model for virion secretion postulates the direct budding of cyoplasmic capsids into ER/Golgi in a nucleus-independent manner. **B** In our proposed model, CRM1-mediated nuclear export of HBc capsids is followed immediately by the microtubule-mediated transport from MTOC at nuclear pore to ER/Golgi for envelopment and virion egress. Both genome-containing and genome-free capsids can undergo nucleocytoplasmic shuttling [24, 26, 29]. Dotted line with a question mark highlights the hypothetical nature of ER/Golgi budding from empty cytoplasmic capsid particles. Nu: nucleus, Cy: cytoplasm

It is generally believed that HBV virion secretion is initiated in the cytoplasm by ER budding from capsid particles containing mature RC DNA genome (Fig. 7A) [66, 67]. However, it cannot be excluded that at least some mature or empty capsids bound for secretion might indirectly reach the perinuclear ER/Golgi en route the nucleus (Fig. 7B; see further discussions below).

In previous studies, HBV 3.5 kb pgRNA can be exported by the NXF1-p15 pathway in a CRM1-independent manner [24, 26]. Two HBc specific NES have been mapped to the arginine-rich domain (ARD) in the cytoplasmic tail of the core protein [24, 25]. As discussed above, the secretion of naked capsids is much less dependent (or nearly independent) on the CRM1 machinery in the SINE-treatment experiment (Fig. 3A). It is likely that most naked capsids can be assembled form core protein in the cytoplasm, which is translated from the non-encapsidated pgRNA exported by the NXF1-P15 route [26]. Recently, we proposed a hypothesis that, in the earlier phase of the HBV life cycle, immature capsids containing an RNA pregenome in the nucleus preferentially takes the CRM1 route for export to the cytoplasm for reverse transcription and DNA synthesis [29]. Despite the fact that a canonical role of CRM1 is mainly in nuclear export, recent reports regarding a potential non-canonical role of CRM1 in adenovirus have been emerging [32, 68, 69]. We report here another unexpected role of CRM1 in HBV virion secretion.

Using the PLA assay, we detected the in vivo interactions between CRM1, HBc, and microtubules (Fig. 6B–D). By confocal microscopy, we also observed colocalization between CRM1, tubulin and HBc protein at the perinuclear region (Additional file 1: Fig. S6). Recently, a cytoplasmic protein Mto1 was shown to dock on the nuclear pore, and facilitate the formation of the non-centrosomal microtubule organizing centers (MTOCs) via interaction with CRM1 and Nup146 FG repeats [70]. By nocodazole treatment, HBc appeared to be accumulated at the perinuclear region around MTOC in hepatocytes [71]. Furthermore, nuclear tubulin was shown to be actively exported by the CRM1 pathway [72]. Therefore, we speculate that HBV virion secretion could be mediated through the CRM1 machinery and the MTOC cytoskeleton.

In our nocodazole experiment using HuH-7 hepatocytes (Fig. 6A), virion secretion was completely abolished by nocodazole at 1–10 μM, yet, the level of intracellular capsids was reduced by only approximately twofold at the same nocodazole concentration. In HepG2-based hepatocytes, nocodazole at 10 μM was found to abrogate the association between HBV core protein and tubulin, resulting in the attenuation of capsid formation [41]. Given the differences in the hepatocyte cell lines and the used nocodazole concentrations, it may not be easy to directly compare our results (Fig. 6A) with those in Iwamoto et al. (2017). Nevertheless, as shown in Fig. 7B, it is tempting to hypothesize that the CRM1 cargo of HBc capsids could first meet microtubule by reaching the MTOC at the nuclear pore. Subsequently, HBc capsids could traffick from nuclear pore to perinuclear ER/Golgi, leading to virion secretion from ER/Golgi to the extracellular compartment in a microtubule-dependent manner. A recent literature reported that HBV exploits ERGIC-53 and COPII in ER/Golgi for egress [23].

CRM1 inhibitors like Xpovio (KPT-330) had been clinically approved in cancer therapeutics [43]. In addition, it has antiviral activities against influenza A virus and respiratory syncytia virus [73, 74]. We demonstrated here that SINE compound could inhibit CRM1-dependent HBV virion secretion. The antiviral potential of CRM1 inhibitors can be further explored in HBV animal models and clinical trials of chronic HBV carriers.

## Conclusions

It is well documented that CRM1 plays a key role in nuclear transport. We described here an unexpected finding that CRM1 is essential to HBV virion secretion containing mature genomes. Recently, we demonstrated that CRM1 inhibitors can interfere with an early event in HBV life cycle by blocking the nuclear export of pgRNA-containing capsids [29]. In this study, we report that CRM1 inhibitors could also interfere with a late event in HBV life cycle by blocking the HBV virion secretion. Promisingly, the dual actions of CRM1 inhibitors against HBV could have a striking “double play” in the baseball game between HBV and chronic carriers. A clinical trial will be warranted for testing its therapeutic potential in the future.

## Supplementary Information

**Additional file 1: Fig S1.** A cartoon illustration for the analysis methods of extracellular and intracellular HBV viral and subviral particles. (A) The extracellular HBV particles in the media were first precipitated by ultracentrifugation through a sucrose cushion before native agarose gel electrophoresis. Viral and subviral particles were separated and characterized by Southern and Western blot analyses. Anti-HBc and anti-HBs antibodies were used consecutively for the same Western blot filters. Southern blot was performed in a separate agarose gel which allows the differentiation of genome-containing (G +) from genome-free (G-) viral and subviral particles. (B) The intracellular core-associated viral RNA and DNA were purified by PEG precipitation and nucleic acid extraction. Then, the RNA/DNA signals were analyzed by Northern blot and Southern blot. 3.5 kb pgRNA: full-length 3.5 kb pre-genomic RNA. RT RNA: reverse transcripted RNA. (Related to Fig. 1. **Fig. S2.** HBc amino acid L68 is required for secretion of naked capsids. (A) Alanine substitutions at L68, L72, V74, L84, V86 and M93 (blue colored) exhibited no effect on HBc nuclear accumulation [29]. (B) Intracellular core-associated RNA and DNA genomes were examined by Northern and Southern blot analyses. Single mutant M93A was defective in RNA packaging and DNA synthesis. Capsid assembly was normal by native agarose gel and Western blot analysis. (C) Extracellular HBV particles were analyzed by the method in Fig. S1A. HBc mutant Single mutant M93A appeared to have lost genome-containing virion secretion. HBc mutant L68A completely lost naked capsid secretion. Both mutant V86A and M93A exhibited strongly decreased signal of genome-containing naked capsids. Red asterisk * indicates reproducibly enhanced signal of secreted virions (see text for discussion). (D) A hexameric version of Fig. 2E. Red: secretion of genome-containing virions. Blue: secretion of naked capsids. Merge: purple. Cyan: a previous map of key residues involved in the secretion of HBV genome-containing virions [37]. (Related to Fig. 2). **Fig S3.** No significant anti-proliferation effect by SINE treatment in HuH-7 cells. Cell proliferation was monitored by (A) alamarBlue and (B) CCK-8 assays after treatment with CRM1 inhibitors. No apparent side effect was detected below 0.25 μM SINE compound. (Related to Fig. 3). **Fig S4.** HBV capsid particles accumulated in the nucleus by SINE compound treatment. Three major patterns of HBc subcellular distribution (Cy > Nu, Nu > Cy, Nu&Cy) were scored [26] in an IFA assay using a rabbit polyclonal anti-HBc antibody (A) and a capsid-specific mouse monoclonal antibody (B). (Related to Fig. 4). **Fig S5.** Cotransfection with a drug-resistant mutant CRM1 rescued HBsAg secretion and shifted nuclear HBc to the cytoplasm in the presence of a SINE compound. (A) In an HBV transient transfection system, HuH-7 cells were treated with or without SINE compound. Cotransfection with a drug-resistant mutant mtCRM1 could significantly rescue the secreted HBsAg by the ELISA assay. (B) Nuclear HBc accumulation induced by the SINE compound treatment can be redirected to the cytoplasm by cotransfection with a drug-resistant mtCRM1 plasmid (see Methods). (C) SINE compound reduced the level of cytoplasmic HBc in HBV transfected HuH-7 cells by subcellular fractionation and Western blot analysis. Cytoplasmic HBc was increased after cotransfection with a drug-resistant mtCRM1 plasmid. (Related to Fig. 5). **Fig S6.** Colocalization of CRM1, microtubule and HBc protein at the perinuclear region by confocal immunofluorescence microscopy. Left panel: Serial sectioning of confocal microscopy was analysed by the ZEISS ZEN Microscope Software Profile. Right panel: Colocalization was revealed by the superimposed colors: tubulin (green), CRM1-mCherry (red) and HBc (cyan) at the perinuclear area. DAPI (blue) (Related to Fig. 6)

## Data Availability

Not applicable.

## Abbreviations

ARD: Arginine-rich domain in HBc
CRM1: Chromosome region maintenance 1, also called exportin1, Xpo1
mtCRM1: Mutant CRM1
Cy > Nu: Predominantly cytoplasmic HBc
ESCRT: Endosomal sorting complex required for transport
G+: Genome-containing
G−: Genome free
HBV: Hepatitis B virus
HBsAg: HBV surface antigen
HBeAg: HBV e antigen
HBc: HBV core protein
HBx: HBV X protein
MTOC: Microtubule organizing center
NES: Nuclear export signals
Nu > Cy: Predominantly nuclear HBc
Nu & Cy: HBc in both nucleus and cytoplasm
pgRNA: Pregenomic RNA
PLA: Proximity ligation assay
Pol: Polymerase:
PRE: Post-transcriptional regulatory element
RC DNA: Double-stranded relaxed-circle DNA
RT RNA: Reverse-transcribed RNAs
SINE: Selective inhibitor of nuclear export
SS DNA: Single-stranded linear DNA
WT-HBV: Wild type HBV
