# Supplementary material for: Hepatitis B virus virion secretion is a CRM1-spike-mediated late event
Source: J Biomed Sci. 2022 Jun 21;29:44. doi: 10.1186/s12929-022-00827-w (PMC9210616; doi:10.1186/s12929-022-00827-w)
Supplement: Supplementary file 1 — Additional file 1: Fig S1. A cartoon illustration for the analysis methods of extracellular and intracellular HBV viral and subviral particles. (A) The extracellular HBV particles in the media were first precipitated by ultracentrifugation through a sucrose cushion before native agarose gel electrophoresis. Viral and subviral particles were separated and characterized by Southern and Western blot analyses. Anti-HBc and anti-HBs antibodies were used consecutively for the same Western blot filters. Southern blot was performed in a separate agarose gel which allows the differentiation of genome-containing (G +) from genome-free (G-) viral and subviral particles. (B) The intracellular core-associated viral RNA and DNA were purified by PEG precipitation and nucleic acid extraction. Then, the RNA/DNA signals were analyzed by Northern blot and Southern blot. 3.5 kb pgRNA: full-length 3.5 kb pre-genomic RNA. RT RNA: reverse transcripted RNA. (Related to Fig. 1. Fig. S2. HBc amino acid L68 is required for secretion of naked capsids. (A) Alanine substitutions at L68, L72, V74, L84, V86 and M93 (blue colored) exhibited no effect on HBc nuclear accumulation [29]. (B) Intracellular core-associated RNA and DNA genomes were examined by Northern and Southern blot analyses. Single mutant M93A was defective in RNA packaging and DNA synthesis. Capsid assembly was normal by native agarose gel and Western blot analysis. (C) Extracellular HBV particles were analyzed by the method in Fig. S1A. HBc mutant Single mutant M93A appeared to have lost genome-containing virion secretion. HBc mutant L68A completely lost naked capsid secretion. Both mutant V86A and M93A exhibited strongly decreased signal of genome-containing naked capsids. Red asterisk * indicates reproducibly enhanced signal of secreted virions (see text for discussion). (D) A hexameric version of Fig. 2E. Red: secretion of genome-containing virions. Blue: secretion of naked capsids. Merge: purple. Cyan: a previous map of key res [file 12929_2022_827_MOESM1_ESM.pdf]

Supplementary Information

Hepatitis B virus virion secretion is a CRM1-spike-mediated late event

Running Title: A hub of sensors for HBV secretion

Authors: Pei-Yi Su[1,2], Shin-Chwen Bruce Yen[1, 2], Ching-Chun Yang[2], Chih-Hsu Chang[2,3], Wen-Chang Lin[2], and Chiaho Shih[1,2*]

1 Graduate Institute of Medicine, Kaohsiung Medical University, Kaohsiung, Taiwan

2 Institute of Biomedical Sciences, Academia Sinica, Taipei, Taiwan

3Graduate Institute of Microbiology, College of Medicine, National Taiwan University, Taiwan

*Lead contact and corresponding author

Mailing address: No.100, Shih-Chuan 1st Road, Sanmin Dist., Kaohsiung City 80708, Graduate Institute of Medicine, Kaohsiung Medical University, Kaohsiung, Taiwan

Tel: 886-7-3121101 ext 2324; E-mail: cshih@kmu.edu.tw

Figure S1

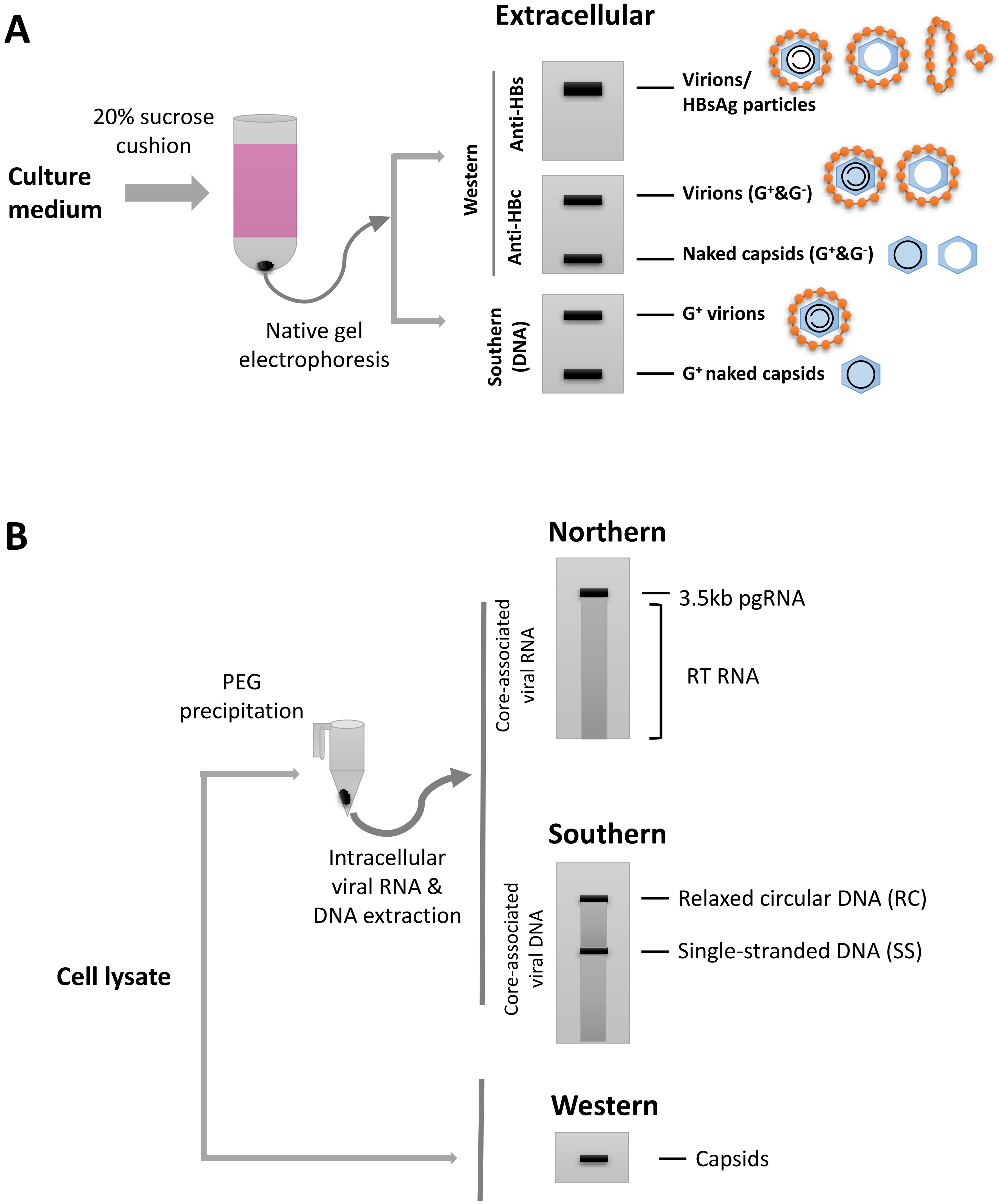
A
Extracellular
20% sucrose
cushion
Culture
medium
Native gel
electrophoresis
Western
Anti-HBs
Anti-HBc
Southern
(DNA)
Virions/
HBsAg particles
Virions (G+&G-)
Naked capsids (G+&G-)
G+ virions
G+ naked capsids
B
Northern
PEG
precipitation
Intracellular
viral RNA &
DNA extraction
Cell lysate
Core-associated
viral RNA
3.5kb pgRNA
RT RNA
Southern
Core-associated
viral DNA
Relaxed circular DNA (RC)
Single-stranded DNA (SS)
Western
Capsids

Figure S2

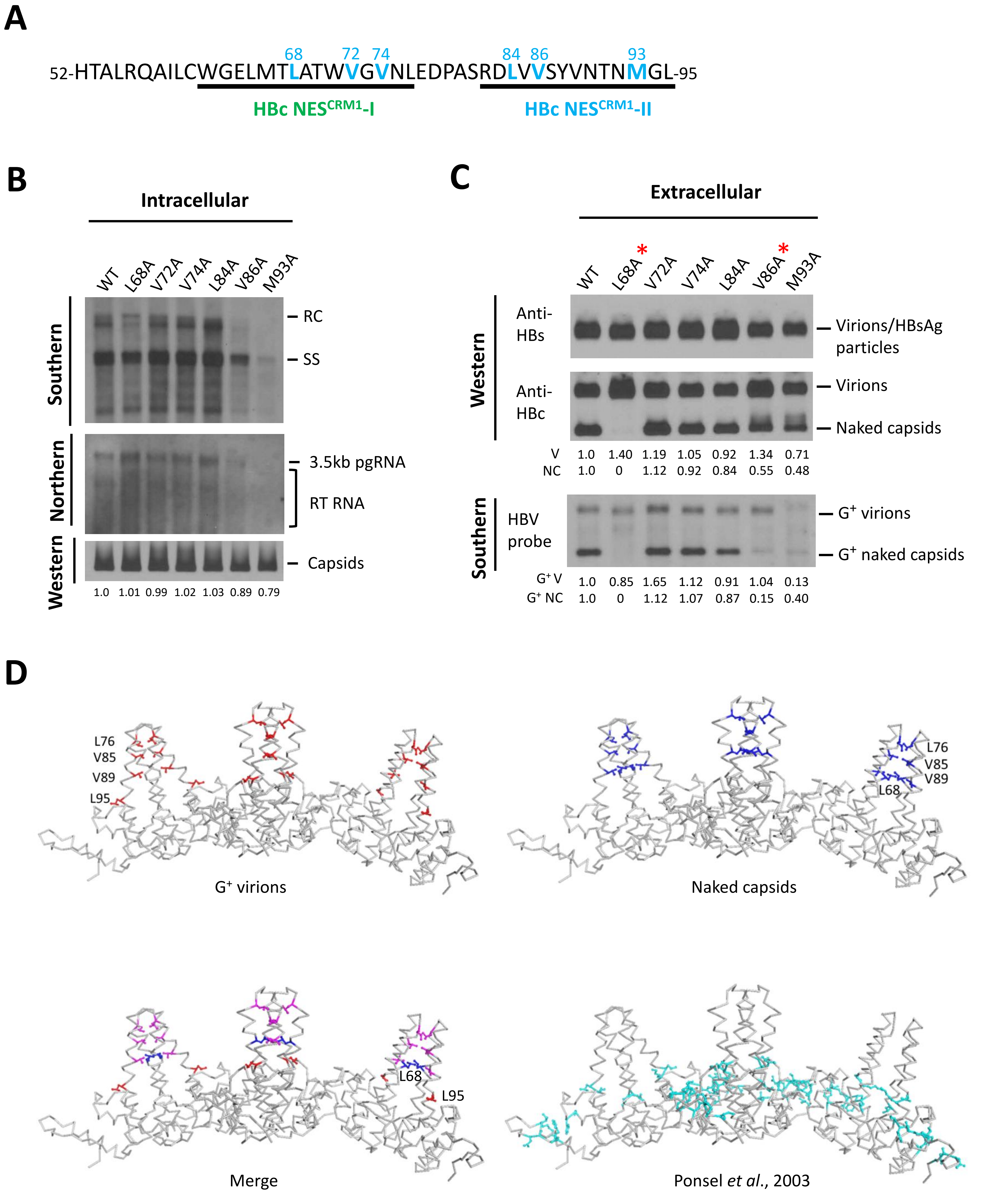
A
68 72 74 84 86 93
52-HTALRQAILCWGELMTLATWVGVNLEDPASRDLVVSYVNTNMGL-95
HBc NESCRM1-I
HBc NESCRM1-II
B
Intracellular
WT L68A V72A V74A L84A V86A M93A
Southern
RC
SS
Northern
3.5kb pgRNA
RT RNA
Western
Capsids
1.0 1.01 0.99 1.02 1.03 0.89 0.79
C
Extracellular
WT L68A * V72A V74A L84A V86A * M93A
Western
Anti-HBs
Virions/HBsAg particles
Anti-HBc
Virions
Naked capsids
V 1.0 1.40 1.19 1.05 0.92 1.34 0.71
NC 1.0 0 1.12 0.92 0.84 0.55 0.48
Southern
HBV probe
G+ virions
G+ naked capsids
G+ V 1.0 0.85 1.65 1.12 0.91 1.04 0.13
G+ NC 1.0 0 1.12 1.07 0.87 0.15 0.40
D
L76
V85
V89
L95
G+ virions
L76
V85
V89
L68
Naked capsids
L68
L95
Merge
Ponsel et al., 2003

Figure S3

A

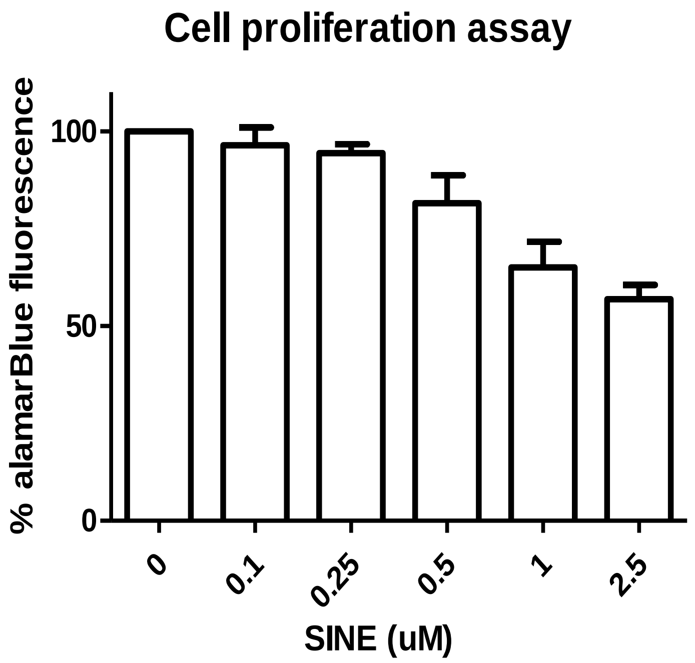

Cell proliferation assay
% alamarBlue fluorescence
100
50
0
0
0.1
0.25
0.5
1
2.5
SINE (uM)

B

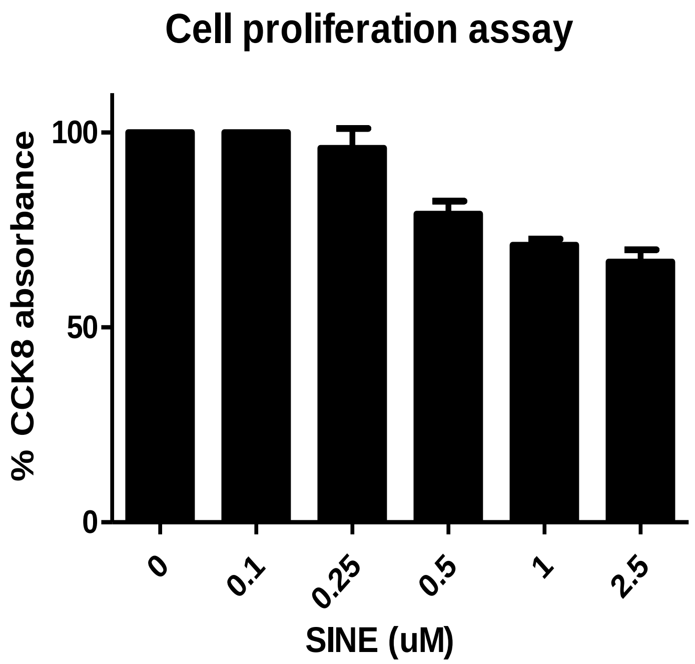

Cell proliferation assay
% CCK8 absorbance
100
50
0
0
0.1
0.25
0.5
1
2.5
SINE (uM)

Figure S4

A

HBc subcellular distribution (rabbit polyclonal Ab)

SINE (µM)	Nu>Cy (%)	Cy>Nu (%)	Nu&Cy (%)	Total cell number scored
**0**	2±0.02	82±0.8	16±0.4	665
**0.1**	12±0.2	54±0.7	34±0.1	374
**0.25**	21±0.1	39±2.6	40±0.4	285
**0.5**	28±1.5	25±3.8	47±0.9	282
**1.0**	29±0.1	20±2.4	51±0.2	282

B

HBV capsid distribution (Mab 3120)

SINE (µM)	Nu>Cy (%)	Cy>Nu (%)	Nu&Cy (%)	Total cell number scored
**0**	11.6±6.9	57.3±5.4	31.1±1.5	628
**0.1**	29±7.9	30±0.69	41±7.2	508
**0.25**	38±6.6	26±8.5	36±1.8	374
**0.5**	42±1.9	26±2.5	33±0.64	223
**1.0**	38.9±0	27.9±0.23	33.2±0.23	198

Figure S5

A

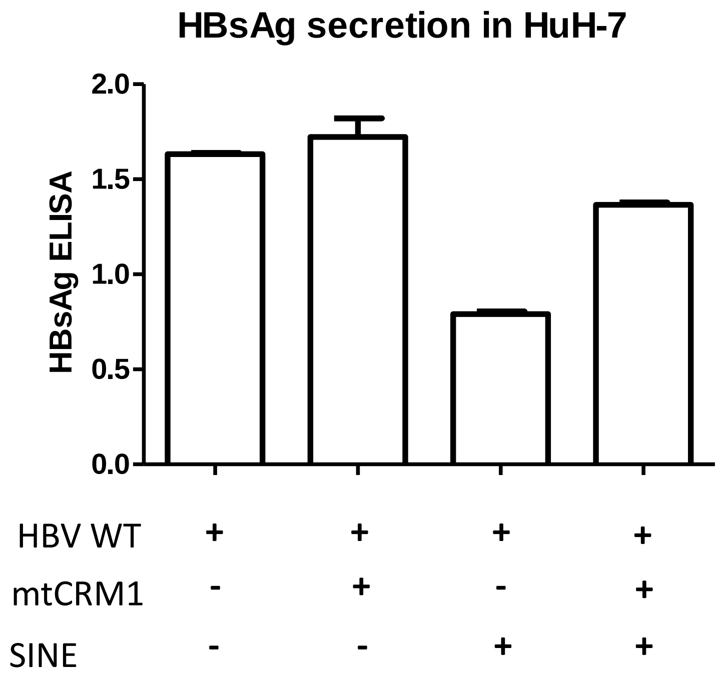

B

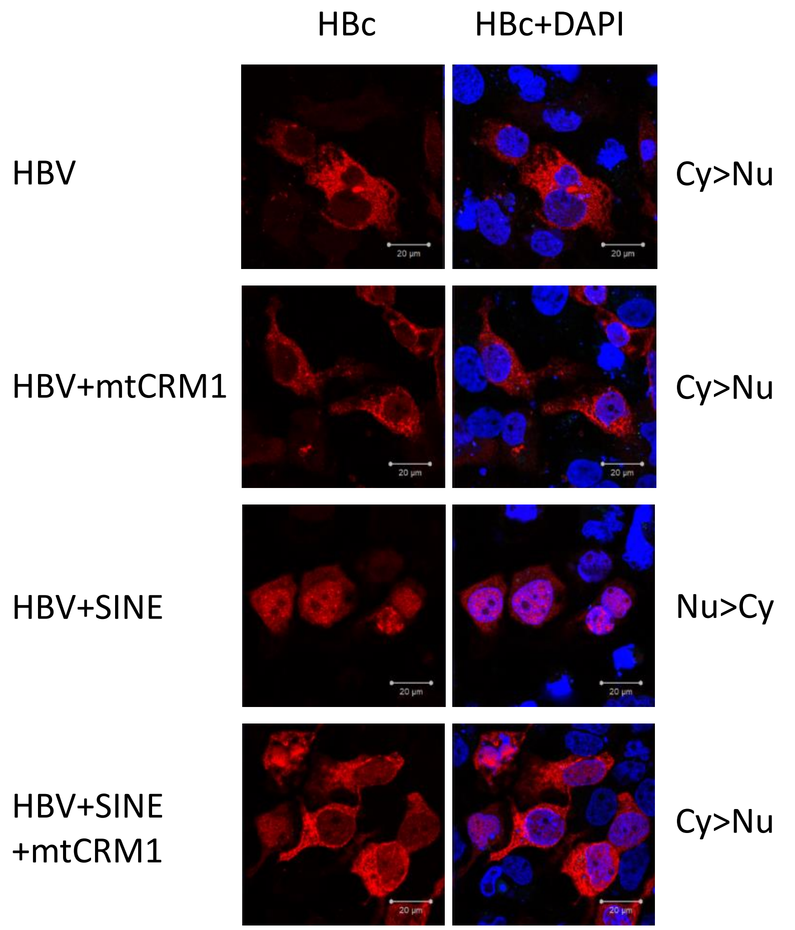

C

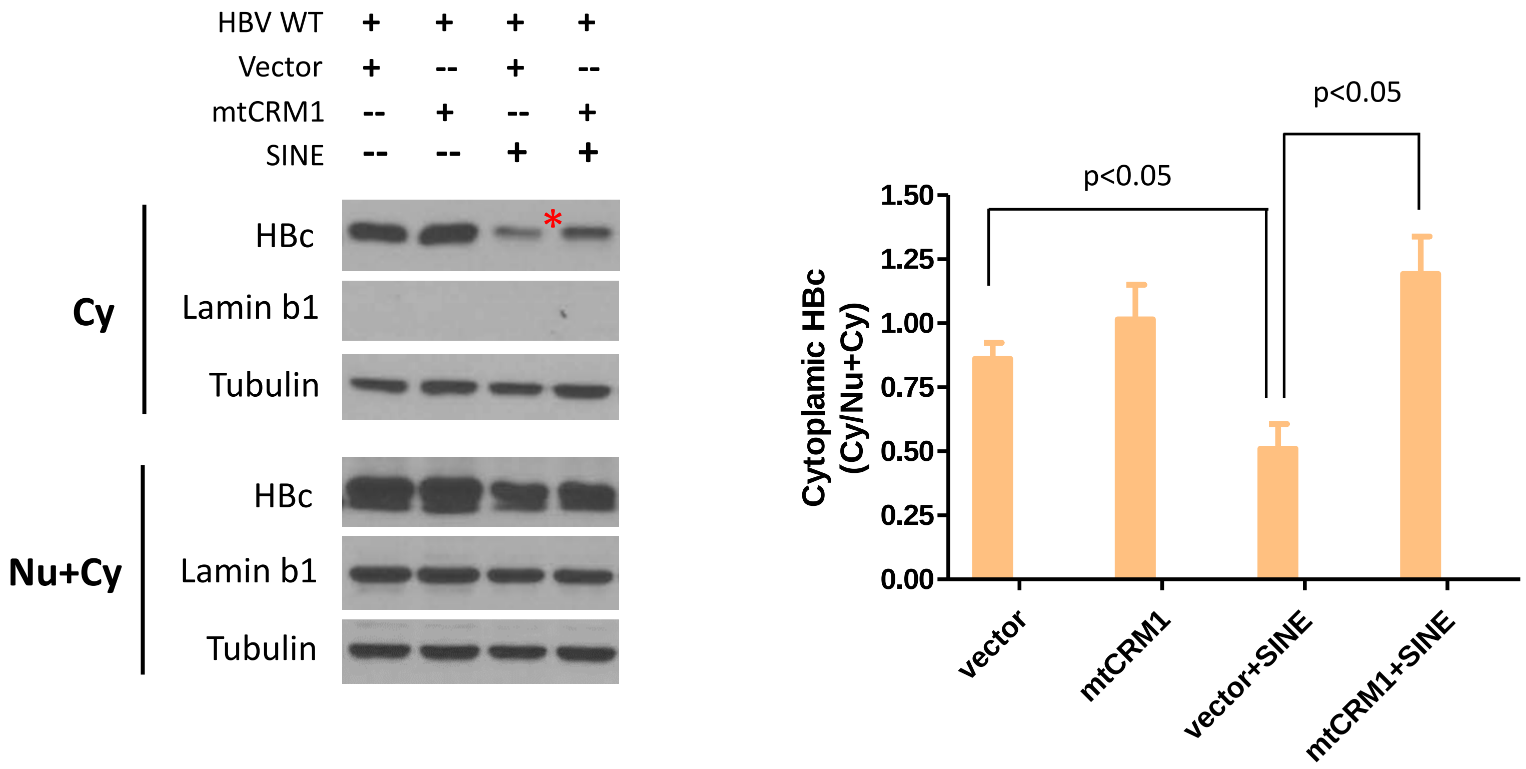

Figure S6

Colocalization of HBc/CRM1/α tubulin in the perinuclear area

Cy Nu Nu Cy

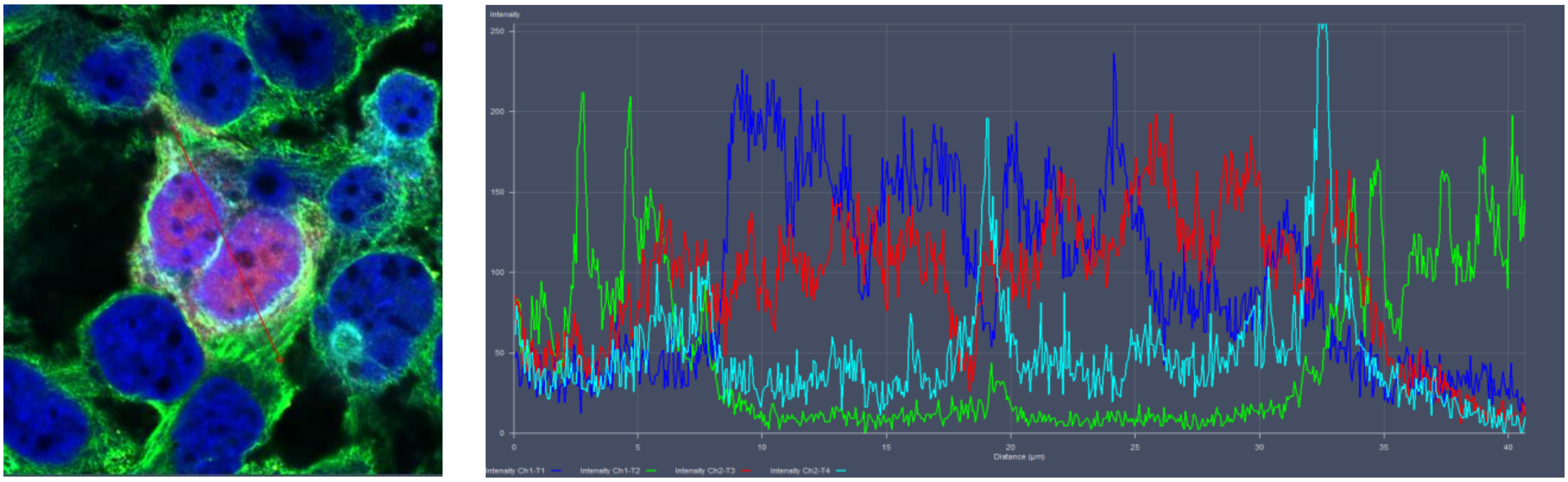

DAPI — α tubulin — CRM1-mCherry — HBc —
